# Pulmonary Injury Secondary to Feeding Tube Misplacement

**Published:** 2014-08-07

**Authors:** Thomas R. Resch, Leigh A. Price, Stephen M. Milner

**Affiliations:** Johns Hopkins Burn Center, The Johns Hopkins University School of Medicine, Baltimore, Md

**Keywords:** CO_2_ monitoring, pulmonary injury, pneumothorax, intensive care, feeding tube

## DESCRIPTION

**Figure 1:**
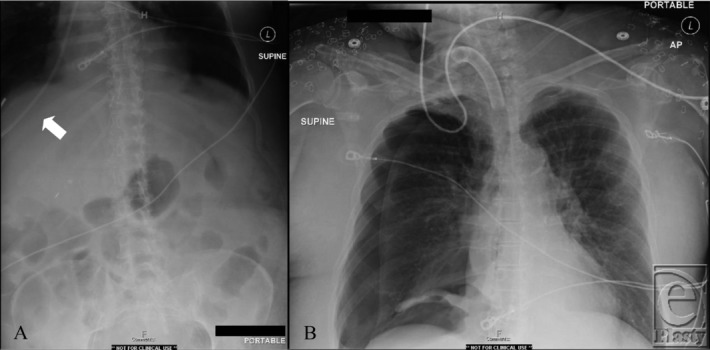
Plain films (*a*) of the abdomen following small bowel feeding tube placement and (*b*) of the chest after immediate removal of the tube.

A 68-year-old female patient was admitted to the intensive care unit with severe burns and inhalation injury. During her hospitalization a nasoenteric small bore feeding tube (SBFT) was placed in a blind fashion at the bedside. Postplacement radiographs were obtained (Fig 1).

## QUESTIONS

1. **What is the diagnosis?**
2. **What is the incidence?**
3. **What are the risk factors?**
4. **What other placement techniques exist?**

## DISCUSSION

**Figure 2:**
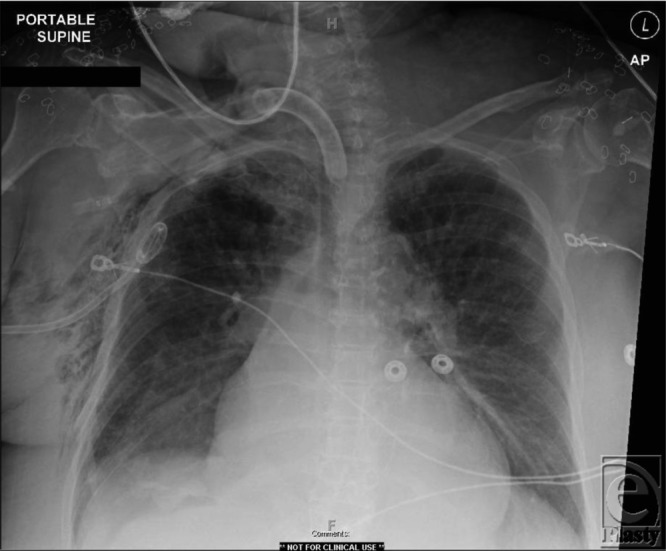
Chest radiograph following decompression of right pneumothorax.

The patient has a large right-sided pneumothorax as a result of feeding tube misplacement into the airway. The tube was removed and a 14 French pigtail catheter was placed to reexpand the lung (Fig 2). A new feeding tube was then inserted by fluoroscopy.

Approximately 1.2% to 2% of all blind SBFT insertions result in airway misplacement, 0.3% to 0.7% cause pulmonary injury (pneumothorax, hemopneumothorax, hydrothorax, empyema, and pneumonia), and 0.1% to 0.3% lead to death.^1^ At first glance, these numbers may seem insignificant, however, if taken in the context of all feeding tube placements in the United States, this would translate into more than 3000 injuries and deaths per year.^1^

Risk factors for SBFT misplacement include altered mental status, sedation, critical illness, intubation (endotracheal or tracheostomy tube), absent cough reflex, difficult or repeat tube placement, noncompliant behavior, and anatomic abnormalities.^1^ Note that the presence of a cuffed tracheal tube is not protective and instead may increase the risk of airway misplacement.^1^^,^^2^ In our case, a tracheostomy tube with an inflated cuff was in place and did not prevent the SBFT from entering the trachea. The tube preferentially went down the airway rather than the esophagus and easily passed the tracheal balloon with little to no resistance when examined fluoroscopically. Finally, greater clinician experience does not appear to decrease misplacements.^1^^,^^2^

Modalities to confirm SBFT placement range from simple bedside maneuvers to advanced technology using electromagnetic imaging. Techniques for bedside assessment include air insufflation during auscultation of the abdomen as well as aspiration of fluid to confirm gastric placement (goal pH < 4).^2^ While fast, simple, and inexpensive, these methods are too inaccurate and unreliable.^2^ The postplacement abdominal radiograph is considered the “gold standard” for confirmation of SBFT position.^3^ However, this will not prevent injury during the insertion process. A 2-step radiographic approach attempts to address this issue with an initial chest radiograph obtained at a SBFT depth of 30 cm to confirm esophageal passage before full advancement of the tube.^4^^,^^5^ This technique can be time consuming and results in additional radiation exposure to the patient. Another adjunct is CO_2_ detection via capnography or colorimetry.^1^^,^^2^ The latter technique has a reported sensitivity and specificity of 100% and may ultimately replace the initial radiograph in the 2-radiograph technique.^1^^-^3 Rarely, false-negative results have been reported secondary to tube obstruction by kinking, mucus, or lubricating jelly.^1^ For this reason, some newer colorimetric detectors employ a small plastic bellows attachment.^1^ Commercially available electromagnetic devices are another tool designed specifically for bedside SBFT placement. These have the advantage of providing real time guidance during the entire placement process via an electromagnetic transmitter in the stylette tip.^1^ This not only helps to avoid injury but may facilitate more precise postpyloric positioning. These devices may ultimately eliminate the need for a confirmatory radiograph.^1^ Drawbacks include the cost of specialized SBFTs and the need for an available device with trained operators.^1^ Finally, fluoroscopy, direct laryngoscopy, and endoscopy are all nearly failsafe placement techniques but are impractical for routine use.^1^

In summary, bronchopulmonary SBFT misplacement is rare but can have significant consequences. The blind placement technique should be limited to noncritically ill patients with normal swallow function. Newer insertion techniques and devices represent safer options with few disadvantages.
